# Two new species of the millipede genus *Glyphiulus* Gervais, 1847 from Laos (Diplopoda, Spirostreptida, Cambalopsidae)

**DOI:** 10.3897/zookeys.722.21192

**Published:** 2017-12-13

**Authors:** Natdanai Likhitrakarn, Sergei I. Golovatch, Khamla Inkhavilay, Chirasak Sutcharit, Ruttapon Srisonchai, Somsak Panha

**Affiliations:** 1 Division of Plant Protection, Faculty of Agricultural Production, Maejo University, Chiang Mai, 50290, Thailand; 2 Institute for Problems of Ecology and Evolution, Russian Academy of Sciences, Leninsky pr. 33, Moscow 119071, Russia; 3 Department of Biology, Faculty of Natural Sciences, National University of Laos, P.O. Box 7322, Dongdok, Vientiane, Laos; 4 Animal Systematics Research Unit, Department of Biology, Faculty of Science, Chulalongkorn University, Bangkok, 10330, Thailand

**Keywords:** Cave, forest, *Glyphiulus*, key, Laos, map, millipede, new species

## Abstract

Two new species of *Glyphiulus* are described and illustrated from northern Laos. The epigean *Glyphiulus subbedosae* Likhitrakarn, Golovatch & Panha, **sp. n.** is the second member of the *granulatus*-group to be found in that country and it seems to be especially similar to *G. bedosae* Golovatch, Geoffroy, Mauriès & VandenSpiegel, 2007. However, it differs from the latter species by a row of several strong setae near the median marginal ridge on the paraprocts, combined with the gnathochilarium being considerably less densely setose on the caudal face, and the anterior gonopods showing a pair of smaller, apical, but larger lateral teeth on the coxosternal plate. *Glyphiulus semicostulifer* Likhitrakarn, Golovatch & Panha, **sp. n.** is the fourth member of the *javanicus*-group to be discovered in Laos, taken from a cave. It seems to be particularly similar to *G. costulifer* Golovatch, Geoffroy, Mauriès & VandenSpiegel, 2007, but is distinguished by the more sparsely alveolate background fine structure of the metazonae, coupled with the gnathochilarium being considerably less densely setose on the caudal face, much stronger paramedian prongs and 4-segmented telopodites on ♂ coxae 1, the slightly longer and more slender apicoparamedian sternal projections on the anterior gonopods, and the much longer flagella of the posterior gonopods. An identification key to and a distribution map of *Glyphiulus* species in Laos are also presented.

## Introduction

The large southeast Asian millipede genus *Glyphiulus* Gervais, 1847 has recently been reviewed and shown to comprise 57 species ranging from southern China, northern Laos, and northern Thailand in the north to southern Vietnam in the south (Golovatch et al. 2007a, b; 2011a, b; Jiang et al. 2017). Only one species, *G. granulatus* (Gervais, 1847), has attained a pantropical distribution due to numerous anthropochore introductions. Golovatch et al. (2007a, b) divide this genus into two species groups, based on the conformation of ♂ legs 1. The *granulatus*-group is distinguished by these legs being devoid of median structures, but supplied instead with two widely separated prongs, coupled with often 1- or 2-segmented telopodites. In contrast, the *javanicus*-group shows ♂ legs 1 provided with medially contiguous, but not entirely fused coxal processes, coupled with usually 4- or 5-segmented telopodites, along with special carinotaxy patterns of the collum and following metaterga.

The Lao People’s Democratic Republic still supports some of the most significant forested areas remaining anywhere in southeast Asia, especially in the mountains in the north and limestone karsts in central parts (Kemp 2011). A distinctive geological feature of the numerous karst landscapes of the country is a multitude of complex cave systems. Such pronounced habitat diversity is also reflected in millipede faunal richness, the diplopod list of Laos being estimated to amount to at least 130 species (Likhitrakarn et al. 2014a).

The first species of *Glyphiulus* to be reported from Laos was *G. bedosae* Golovatch, Geoffroy, Mauriès & VandenSpiegel, 2007, a cave-dweller described from Tham Pha Kouang Cave, Nong Kiaw (Muang Ngoy), Luang Prabang Province, representing the *granulatus*-group (Golovatch et al. 2007a). Almost simultaneously, a further three new species from the *javanicus*-group were added: *G. costulifer* Golovatch, Geoffroy, Mauriès & VandenSpiegel, 2007, from Tham Pha Kouang Cave, Nong Kiaw (Muang Ngoy), Luang Prabang Province, *G. subcostulifer* Golovatch, Geoffroy, Mauriès & VandenSpiegel, 2007, from Tham None Cave, Vang Vieng, Vientiane Province, and *G. percostulifer* Golovatch, Geoffroy, Mauriès & VandenSpiegel, 2007, from Tham Thê Cave, Ban Nakok (Nakhok), Khammouan Province (Golovatch et al. 2007b). All four species seem to be troglophilic and endemic to Laos (Fig. 8).

Since the latest catalogue of the Diplopoda of Laos which listed 34 species (Likhitrakarn et al. 2014a), another 29 new species have been added (Likhitrakarn et al. 2014b, 2014c, 2015a, 2015b, 2016a, 2016b; Golovatch 2016a, 2016b; Golovatch et al. 2016a, 2016b; Liu et al. 2017a). Yet neither new *Glyphiulus* species nor records have since been documented from Laos.

The present paper is devoted to descriptions of two new species of *Glyphiulus* from Laos, coupled with a distributional map of and a key to all six species of the genus currently known to occur in that country.

## Materials and methods

New material was collected from northern Laos in 2014 by SP and members of the Animal Systematics Research Unit, Chulalongkorn University. Photographs of live animals were taken in the laboratory using a Nikon 700D digital camera with a Nikon AF-S VR 105mm macro lens. Specimens were preserved in 75% ethanol, and morphological observations made under an Olympus SZX7stereo microscope.

Scanning electron micrographs (SEM) were taken with a JEOL, JSM–5410 LV microscope, and the material returned to alcohol upon examination. Pictures of one of the gonopods of the holotypes were taken in the laboratory and assembled using the “Cell^D^” automontage software of the Olympus Soft Imaging Solution GmbH package. The key to all species is principally based on the descriptions by Golovatch et al. (2007a, 2007b, 2010, 2011a, 2011b). The holotypes, as well as most of the paratypes are housed in the Museum of Zoology, Chulalongkorn University (**CUMZ**), Bangkok, Thailand; paratypes have been donated to the collection of the Zoological Museum, State University of Moscow, Russia (**ZMUM**), as indicated in the text.

The collecting sites were located by GPS using the WGS84 datum.

The carinotaxy formulae in the descriptions follow those in Golovatch et al. (2007a, 2007b), while body segment counts are after Enghoff et al. (1993).

## Taxonomic part

### Family Cambalidae Cook, 1895

#### Genus *Glyphiulus* Gervais, 1847

##### Glyphiulus subbedosae

Taxon classificationAnimaliaSpirostreptidaGlyphiulidae

Likhitrakarn, Golovatch & Panha sp. n.

http://zoobank.org/E7D45B2D-E78C-43BE-B6F6-153DE3846F06

**Figure 1.**
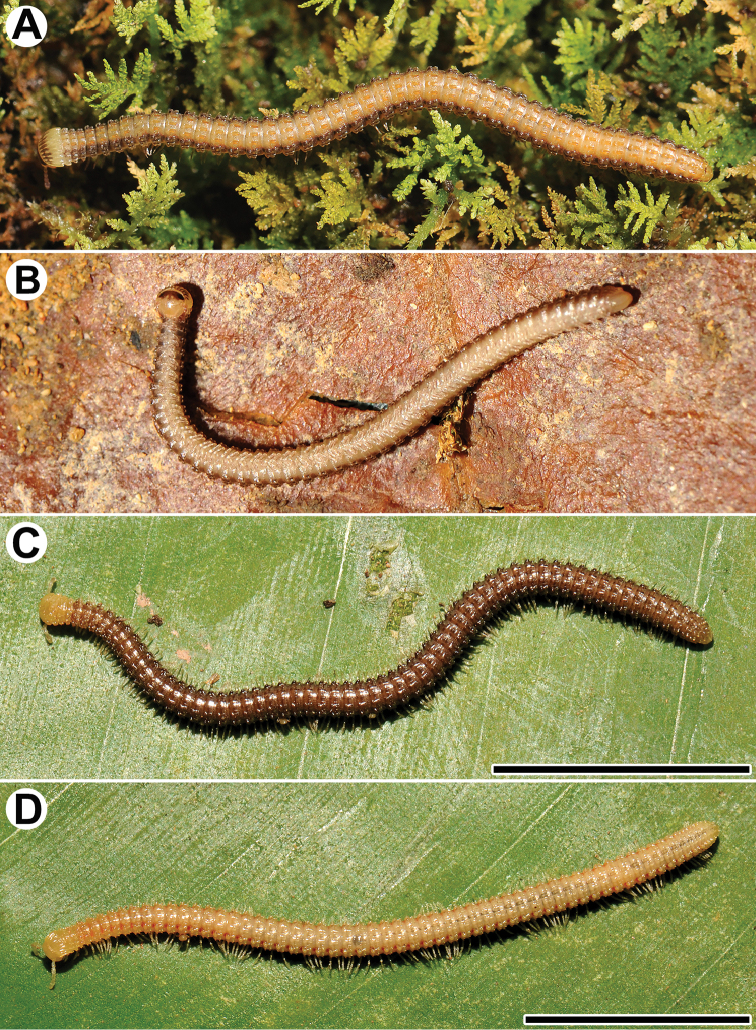
Habitus, live coloration. **A, B** *Glyphiulus subbedosae* sp. n., ♀ paratype from Kacham Waterfall, depicted not to scale **C, D** *Glyphiulus semicostulifer* sp. n., ♀ paratype. Scale bars: 10 mm.

**Figure 2.**
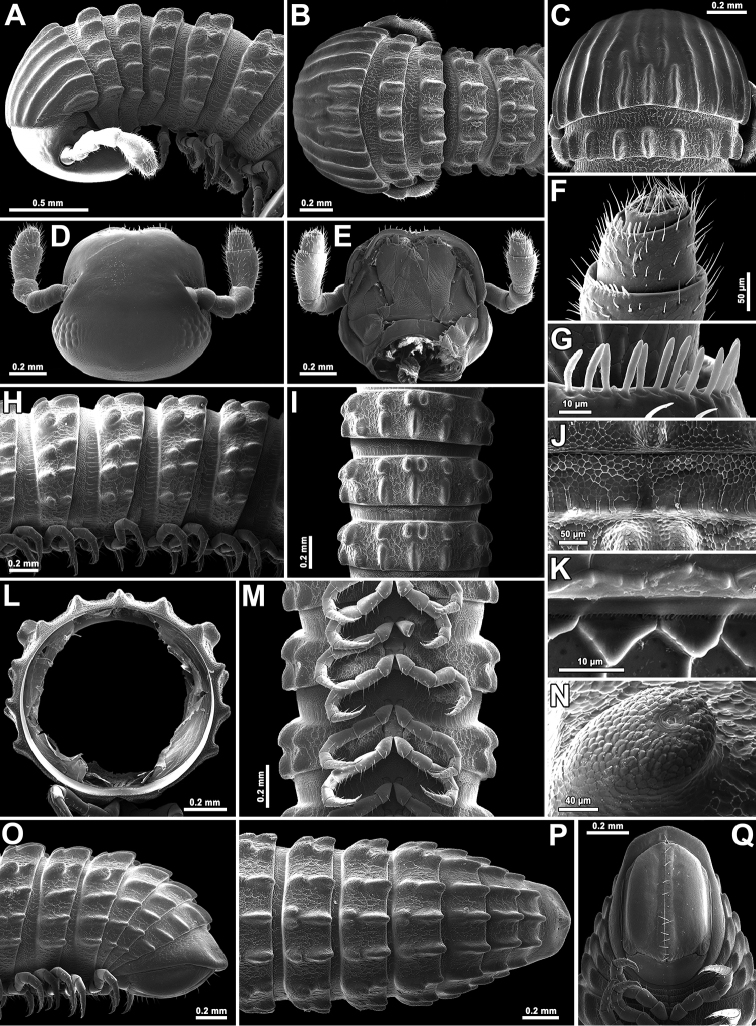
*Glyphiulus subbedosae* sp. n., **A–C, H–Q** ♀ paratype from Kacham Waterfall **D–G** ♂ holotype **A, B** anterior part of body, lateral and dorsal views, respectively **C** collum and body segment 2, dorsal view **D** cephalic capsule, dorsal view **E** gnathochilarium, ventral view **F** apical part of antenna, ventral view **G** bacilliform sensilla on antennomere 5, lateral view **H, I, M** midbody segments, lateral, dorsal and ventral views, respectively **J** midbody prozonite enlarged, dorsal view **K** limbus **L** cross-section of midbody segment **N** enlarged ozopore region, lateral view **O–Q** posterior part of body, lateral, dorsal and ventral views, respectively.

**Figure 3.**
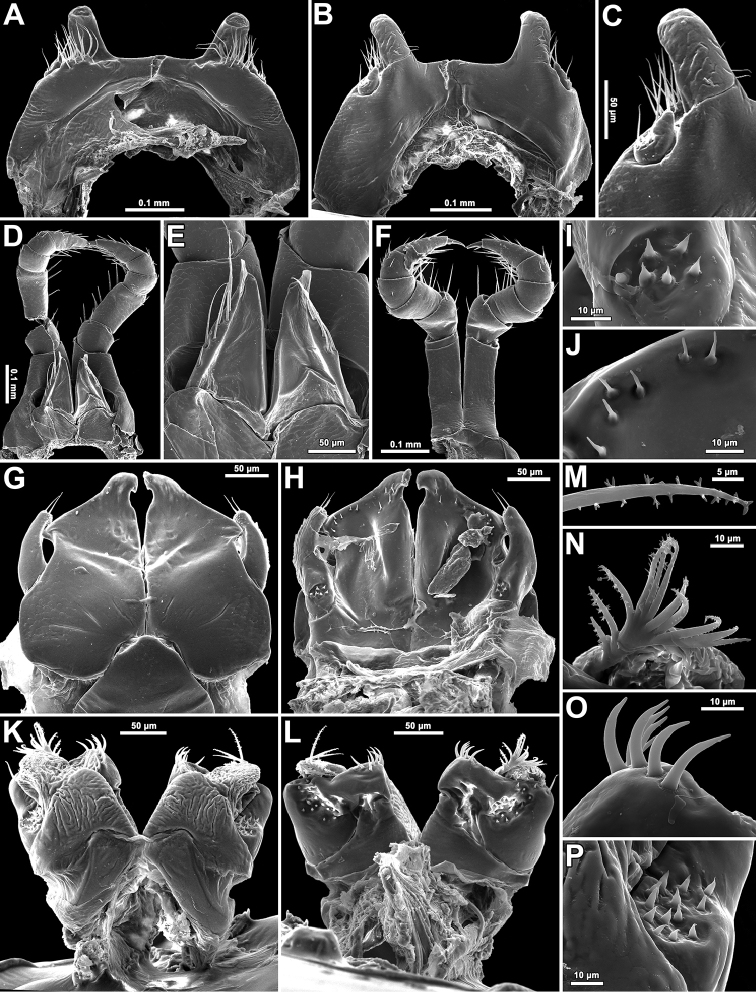
*Glyphiulus subbedosae* sp. n., ♂ holotype. **A, B** legs 1, caudal and frontal views, respectively **C** leg 1, frontal view **D** legs 2, caudal view **E** penes, caudal view **F** legs 3, frontal view **G, H** anterior gonopods, frontal and caudal views, respectively **I** microsetae at base of telopodite of anterior gonopod **J** microsetae on posterior coxosternum of anterior gonopod **K, L** posterior gonopods, frontal and caudal views, respectively **M** plumose seta on flagellum **N** distal part of flagellum of posterior gonopod **O** median lobe of posterior gonopod **P** microsetae at base of posterior gonopod, caudal view.

**Figure 4.**
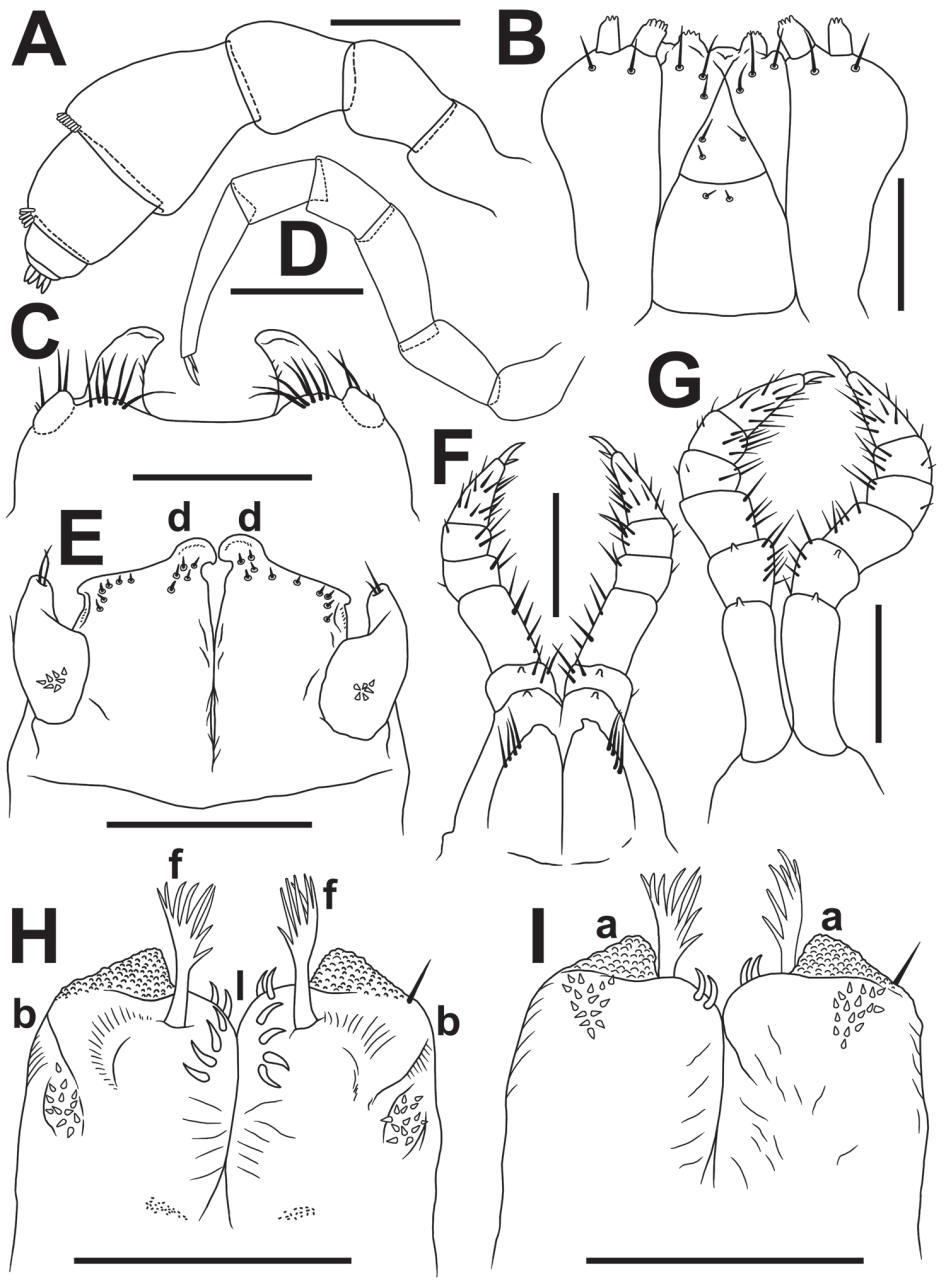
*Glyphiulus subbedosae* sp. n., ♂ paratype from small waterfall near road. **A** antenna, lateral view **B** gnathochilarium, ventral view **C** legs 1, frontal view **D** midbody leg **E** anterior gonopods, caudal view **F** legs 2, caudal view **G** legs 3, caudal view **H, I** posterior gonopods, caudal and frontal views, respectively. Scale bars: 0.2 mm.

Figs 1A, B , 2 , 3 , 4

###### Type material.

Holotype ♂ (CUMZ), Laos, Luang Prabang Province, Chomphet District, Kacham Waterfall, 442 m a.s.l., 19°38'57"N, 100°04'52"E, 30.08.2014, leg. C. Sutcharit and R. Srisonchai.

Paratypes. 4 ♂, 7 ♀ (CUMZ), 1 ♂, 2 ♀ (ZMUM), same locality, together with holotype. 4 ♂, 1 ♀ (CUMZ), same District, small waterfall near road, 405 m a.s.l., 19°41'54"N, 102°07'52"E, 01.07.2014, leg. R. Srisonchai.

###### Etymology.

To emphasize the obvious similarities to *G. bedosae* Golovatch, Geoffroy, Mauriès & VandenSpiegel, 2007.

###### Diagnosis.

This new species is particularly similar to *G. bedosae*, with which it shares the following diagnostic characters: the presence of a row of several strong setae near the median marginal ridge on the paraprocts, combined with the gnathochilarium being considerably less densely setose on the caudal face, and the anterior gonopods showing a pair of smaller apical. It differs from *G. bedosae* primarily by the larger lateral teeth on the coxosternal plate. See also Key below.

###### Description.

Length of holotype ca 12.1 mm; that of paratypes 12.1–23.1 (♂) or 12.2–18.3 mm (♀); midbody segments round in cross-section (Fig. 2L), their width (horizontal diameter) and height (vertical diameter) similar, width of holotype 1.0 mm, of paratypes 0.8–1.5 (♂) or 0.9–1.2 mm (♀).

*Coloration* of live animals brownish yellow (Fig. 1A, B); unfaded specimens variegated, with contrasting dark brownish, lateral, longitudinal stripes above ozopores on each side, both interrupted mid-dorsally by a light wide axial stripe; vertex dark brown, anterior half of collum blackish to dark brown; venter, legs and telson light yellowish to brownish yellow; ocellaria blackish; coloration in alcohol similar, but body brownish yellow to light brownish; vertex dark brown to brown, anterior halves of both collum and head light brown to dark brown; eyes blackish to brownish.

*Body* with 50p+2a+T segments (holotype); paratypes with 39–58p+2(3)a+T (♂) or 41–47p+2–4a+T (♀) segments. Eye patches transversely ovoid, each composed of 10–18 rather flat ocelli in 4 or 5 irregular longitudinal rows (Fig. 2D). Antennae short and clavate (Figs 1A, 2A, D, E), extending behind segment 3 laterally, antennomeres 5 and 6 each with a small distoventral group or corolla of bacilliform sensilla (Fig. 2F, G). Gnathochilarium with a clearly separated promentum (Figs 2E, 4B).

*Head* width = segment 2 < collum = midbody segment (close to 13th to 15th) > segment 3 = 6 > 4 < 5 < 7 < 8 = 10; body abruptly tapering towards telson on a few posteriormost segments (Fig. 2P). Postcollar constriction very evident (Fig. 2B).

*Collum* with 7+7 longitudinal crests starting from anterior edge, but both median crests interrupted in about caudal 1/2–1/3, being replaced there by similar 1+1+1 crests; carinotaxy formula 1–6+7a+pc+ma (Fig. 2B, C).

Following *metaterga* similarly strongly crested (Figs 1A, 2A–C, H, I, O, P), especially from segment 5 on, whence enlarged porosteles commence, these becoming completely absent from legless segments due to loss of ozopores (Fig. 2P). Porosteles large, conical, round, directed caudolaterad, wider than high (Fig. 2N). Midway metatergal crests on segment 5 distinctly divided into two at about 1/3 of metatergal height, each half rather evident and well rounded, nearly undivided and small tubercles in their stead in legless segments in front of telson (Fig. 2B, I, O, P). Carinotaxy formulae 3+I+4+I+3 and 3+i+3+i+3, the former standing for frontal row of crests, the latter for caudal one, both fairly independent (Fig. 2A–C, H, I, O, P).

*Tegument* extremely delicately and quite sparsely alveolate-areolate (Fig. 2K), dull throughout. Fine longitudinal striations in front of stricture between pro- and metazona, remaining surface of prozona very delicately shagreened (Fig. 2J). Metatergal setae absent. Segments 2 and 3 each with long pleural flaps. Limbus extremely finely and regularly spiculate (Fig. 2K). Epiproct (Fig. 2O–Q) simple, regularly rounded caudally, faintly convex medially. Paraprocts regularly convex, each with premarginal sulci medially and with a row of several strong setae at medial margin (Fig. 2Q). Hypoproct transversely bean-shaped, slightly concave caudally, with 1+1 strongly separated setae near caudal margin (Fig. 2Q).

*Ventral flaps* behind gonopod aperture on ♂ segment 7 barely distinguishable as low swellings forming no marked transverse ridge.

*Legs* short, on midbody segments about 2/3 length of body height (Figs 2A, H, M, O, 4D). Claw at base with a strong accessory spine almost half as long as main claw (Fig. 4D). Tarsi and tarsal setae very delicately fringed.

♂ legs 1 highly characteristic (Figs 3A–C, 4C) in being very strongly reduced, represented only by a sternum devoid of any median or paramedian structures, but carrying 1+1 strongly separated prongs, both evidently curved posteriad and bearing several strong setae and rudimentary, 2-segmentel leg vestiges at base on caudal face (Figs 3B, C, 4C).

♂ legs 2 very slightly hypertrophied (Figs 3D, E, 4F), only claw and, anteriorly, coxa somewhat reduced; penes broad, oblong-subtrapeziform, each with 4–6 strong setae distolaterally (Figs 3D, E, 4D).

♂ legs 3 modified in having coxa especially slender and elongate (Figs 3F, 4G).

Anterior *gonopods* (Figs 3G–J, 4E) with a typical shield-like coxosternum which is rather sparsely microsetose on caudal face (Fig. 3H, J) and shows its inner, somewhat elevated, axe-shaped processes, as well as small, but obvious, apicolateral teeth. Telopodite small, but movable, 1-segmented, lateral in position, with 2 or 3 strong apical setae and a field of microsetae at base (Fig. 3H, I), modestly higher than adjacent lateral corner of coxosternum.

Posterior gonopods (Figs 3K–P, 4H, I) compact, broadly subquadrate, coxite medio-apically with a long, plumose, apical flagellum (f) (Fig. 3M, N) with evident spikes paramedially (Fig. 3K–O); caudal piece (telopodite) (b) microsetose laterally, both b and strongly setose lobes (l), lower than frontal, microsetose, median piece (a).

###### Remarks.

The *granulatus*-group currently encompasses 34 described species. The above new one is only the second species in this group to be reported from Laos. Two populations have been found, each from near a forest at a waterfall, and both show the remarkable colour pattern as described above.

##### Glyphiulus semicostulifer

Taxon classificationAnimaliaSpirostreptidaGlyphiulidae

Likhitrakarn, Golovatch & Panha sp. n.

http://zoobank.org/6FCD27DF-7566-42F4-92D5-63F6EDDBA66B

**Figure 5.**
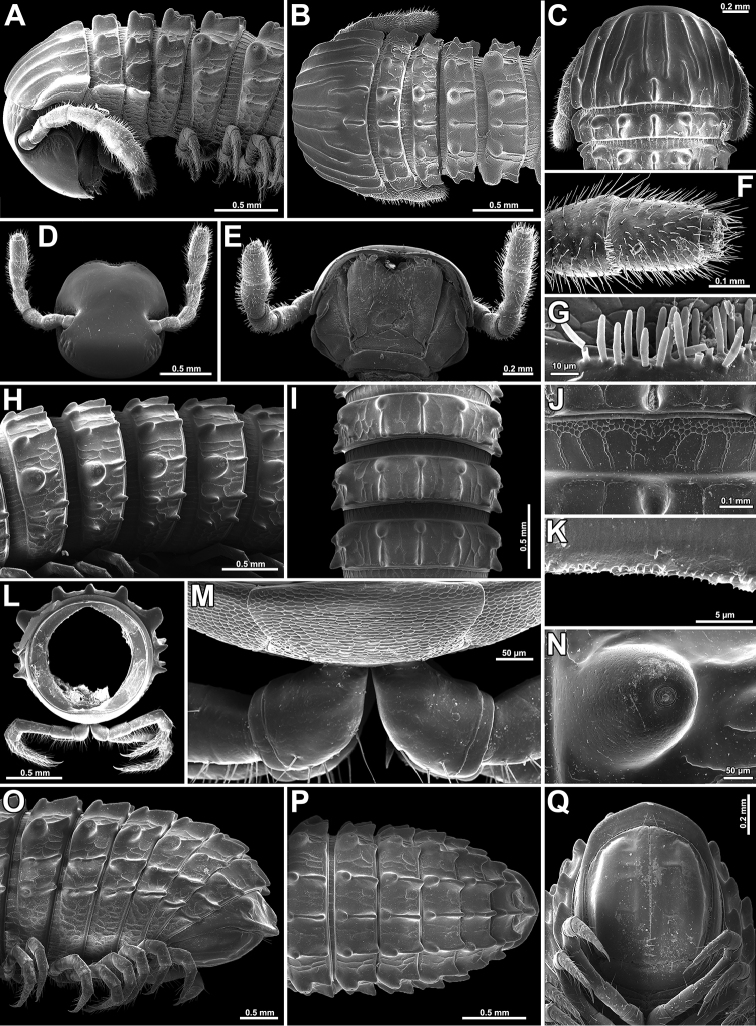
*Glyphiulus semicostulifer* sp. n., **A–C, H–Q** ♀ paratypes **D–G** ♂ paratype **A–B** anterior part of body, lateral and dorsal views, respectively **C** collum and body segments 2 and 3, dorsal view **D** cephalic capsule, dorsal view **E** gnathochilarium, ventral view **F** apical part of antenna, ventral view **G** bacilliform sensilla on antennomere 5, lateral view **H, I** midbody segments, lateral and dorsal views, respectively **J** midbody prozona enlarged, dorsal view **K** limbus, dorsal view **L** cross-section of midbody segment **M** midbody sternite and coxae, frontal view **N** porostele, lateral view **O–Q** posterior part of body, lateral, dorsal and ventral views, respectively.

**Figure 6.**
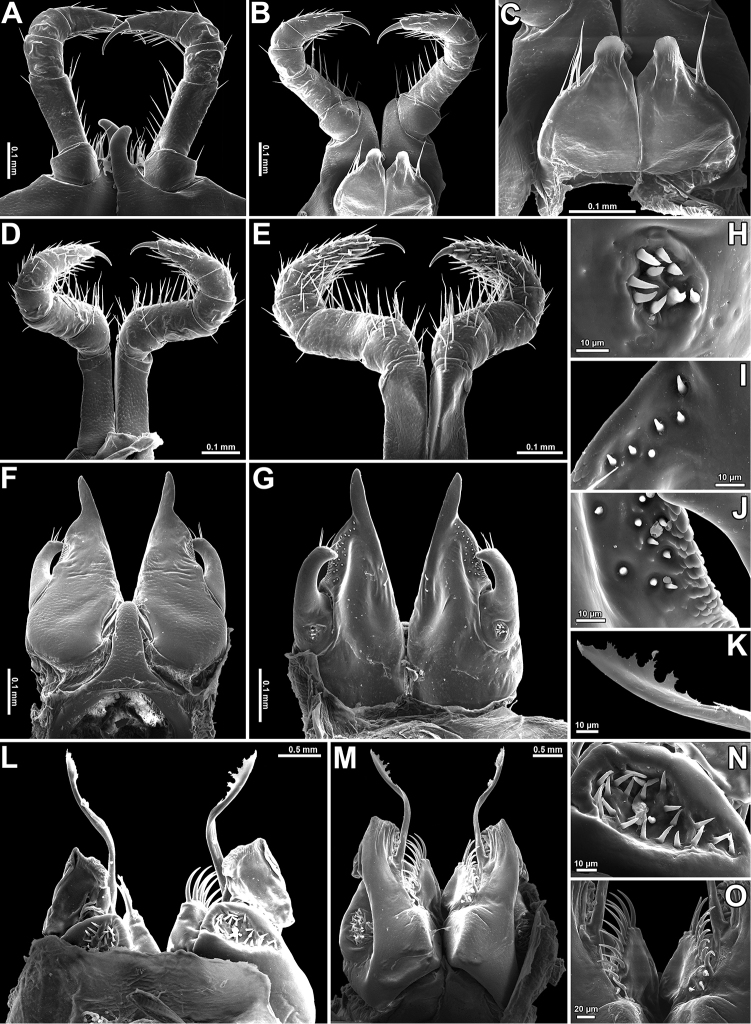
*Glyphiulus semicostulifer* sp. n., ♂ paratype. **A** legs 1, frontal view **B** legs 2, caudal view **C** penes, caudal view **D, E** legs 3, caudal and frontal views, respectively **F, G** anterior gonopods, frontal and caudal views, respectively **H** microsetae at base of telopodite on anterior gonopod **I, J** microsetae on posterior coxosternum of anterior gonopod **L, M** posterior gonopods, caudal and frontal views, respectively **K** distal part of flagellum of posterior gonopod **N** microsetae at base of posterior gonopod, frontal view **O** paramedian lobes on posterior gonopods.

**Figure 7.**
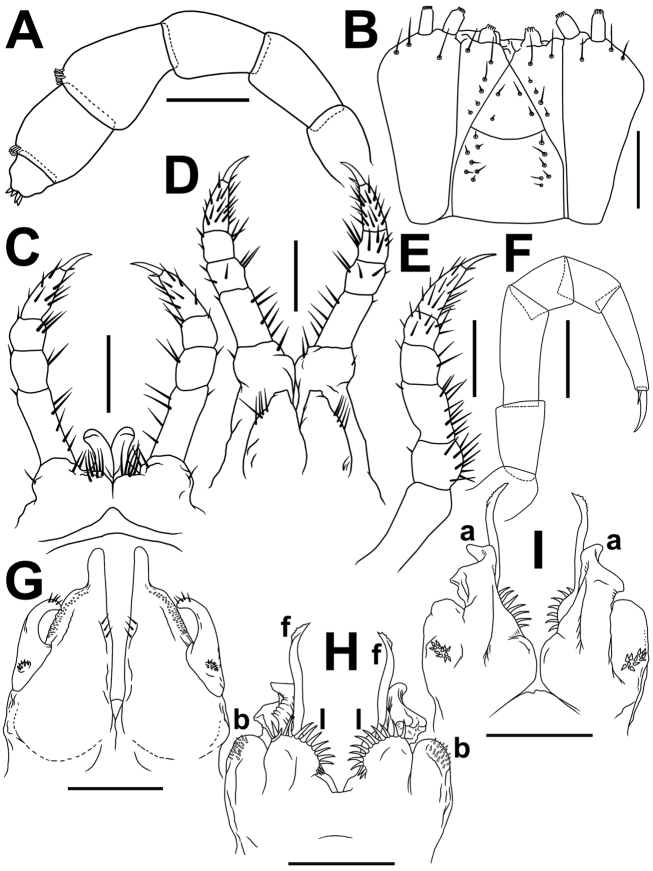
*Glyphiulus semicostulifer* sp. n., ♂ holotype. **A** antenna, lateral view **B** gnathochilarium, ventral view **C** legs 1, frontal view **D** legs 2, caudal view **E** leg 3, caudal view **F** midbody leg **G** anterior gonopods, caudal view **H, I** posterior gonopods, frontal and caudal views, respectively. Scale bars: 0.2 mm.

Figs 1C, D , 5 , 6 , 7

###### Type material.

Holotype ♂ (CUMZ), Laos, Luang Namtha Province, Viengphoukha District, Kao Rao Cave, 737 m a.s.l., 20°43'30"N, 101°09'04"E, 12.10.2014, leg. C. Sutcharit and R. Srisonchai.

Paratypes: 6 ♂, 16 ♀, 8 juveniles (CUMZ), 1 ♂, 2 ♀ (ZMUM), same locality, together with holotype.

###### Etymology.

To emphasize the obvious similarities to *G. costulifer* Golovatch, Geoffroy, Mauriès & VandenSpiegel, 2007.

###### Diagnosis.

This new species is particularly similar to *G. costulifer*, with which it shares the following diagnostic characters: the unique carinotaxy formulae, coupled with anterior gonopod structural details. It differs from *G. costulifer* by the more sparsely alveolate background fine structure of the metazonae, coupled with the gnathochilarium being considerably less densely setose on the caudal face, the paramedian coxal prongs on ♂ legs 1 much stronger and their telopodites 4-segmented, the apicoparamedian sternal projections on the anterior gonopods slightly longer and more slender, and the flagella of the posterior gonopods much longer. See also Key below.

###### Description.

Length of holotype ca 19.4 mm; adult paratypes 17.2–25.3 (♂) or 17.5–25.6 mm long (♀), juveniles 1.34–1.79 mm long; midbody segments round in cross-section (Fig. 5L), their width (horizontal diameter) and height (vertical diameter) similar, width in holotype 1.4 mm; paratypes 1.1–1.5 (♂), 0.9–1.5 (♀) or 0.9–1.1 mm (juveniles).

*Coloration* of live animals dark brown to red-brownish (Fig. 1C), with contrasting light yellow head, antennae, collum, segments 2 and 3, sometimes segment 4 as well; telson yellow-brown, venter and legs brownish yellow to brownish red, ocellaria blackish, lateral longitudinal stripes above porosteles brownish; a thin axial line traceable due to darker median crests, sometimes body uniformly yellowish to brownish yellow (Fig. 1D); coloration in alcohol, after three years of preservation similar, but telson light brownish, venter and legs brownish yellow to brownish, lateral longitudinal stripes brownish to brownish red.

*Body* with 52p+4a+T (holotype); paratypes with 52–67p+2–4a+T (♂), 49–60p+2–4a+T (♀) or 41–50+5a+T (juveniles). Eye patches transversely ovoid, each composed of 7–11 blackish, rather flat ocelli in 4 or 5 irregular longitudinal rows (Fig. 5D). Antennae short and clavate (Figs 1C, D, 5A–E, 7A), extending behind segment 3 laterally, antennomeres 5 and 6 each with a small distoventral group or corolla of bacilliform sensilla (Fig. 5F, G). Gnathochilarium with a clearly separated promentum (Figs 5E, 6B, 7B).

*Head* width = segment 2 < collum = midbody segment (close to 8^th^ to 10^th^) > segment 3 = 5 > 4 < 7 < 8 = 10; body abruptly tapering towards telson on a few posteriormost segments (Fig. 5P). Postcollar constriction very evident (Fig. 5B).

*Collum* nearly smooth, carinotaxy formula 1+2c+3–4+5c+6a+pc+ma (Fig. 5A–C), with 6+6 longitudinal crests starting from anterior edge, but both median crests interrupted in about caudal 2/3–3/4, being replaced there by similar 1+1+1 crests.

Following *metaterga* similarly strongly crested (Figs 1C, D, 5A–C, H, I, O, P), especially from segment 5 on, whence porosteles commence (Fig. 5A, B), smaller tubercles in their stead on legless segments in front of telson due to loss of ozopores (Fig. 5P). Porosteles large, conical, round, directed caudolaterad, wider than high (Fig. 5N), ozoporiferous crests distinctly divided into two about midway, their frontal halves being higher (Fig. 5A, B, H, I, O, P). Carinotaxy formulae 2+I/i+3/3+I/i+2 on segments 2–3, as well as on the last 1–2 leg-bearing and legless segments (Fig. 5A, B, O, P); midbody segments showing all dorsal crests subdivided transversely (carinotaxy formulae 2/2+I/i+3/3+I/i+2/2) and sharper, especially so lateral crests (Fig. 5H, I).

*Tegument* delicately alveolate-areolate (Fig. 5A, B, H–J, M, O, P), dull throughout. Fine longitudinal striations in front of stricture between pro- and metazona, and remaining surface of prozona very delicately shagreened (Fig. 5J). Metatergal setae absent. Segments 2 and 3 with long pleural flaps. Limbus extremely finely and more or less regularly denticulate (Fig. 5K). Epiproct (Fig. 5O, P) simple, with an evident lateral tubercle placed level with ozoporiferous crests, also with a rounded ridge in caudal part and an evident axial rib dorsally. Paraprocts rather regularly convex, each with a faint premarginal sulcus medially (Fig. 5Q). Hypoproct bean-shaped, with 1+1 strongly separated setae near caudal margin (Fig. 5Q).

*Ventral flaps* behind gonopod aperture on ♂ segment 7 rather well distinguishable as low swellings forming a bare transverse ridge.

*Legs* rather short, on midbody segments about 3/4 length of segment height (Figs 5A, L, O, 7F). Claw at base with a strong accessory spine about 1/3–1/4 length of main claw. Tarsi and tarsal setae very delicately fringed (Fig. 7F).

♂ legs 1 highly characteristic (Figs 6A, 7C) in showing nearly fully developed, 4-segmented telopodites and a pair of large, subdigitiform, medially contiguous, but apically diverging coxal processes with a groups of long and strong setae at base.

♂ legs 2 nearly normal, only claw and, anteriorly, coxa somewhat reduced, and femur abbreviated on frontal face; penes broad, rounded, each with 4–5 strong setae distolaterally (Figs 6B, C, 7D).

♂ legs 3 modified in having coxa especially slender and elongate (Figs 6D, E, 7E).

Anterior *gonopods* (Figs 6F–J, 7G) with a typical shield-like coxosternum, this being rather densely microsetose on caudal face (Fig. 6G, I, J), with a high, digitiform, apicomesal process (d). Telopodite typical, slender, movable, 1-segmented, lateral in position, with 3–5 strong apical setae and a field of microsetae at base (Fig. 6H).

Posterior gonopods (Figs 6K–O, 7H, I) highly compact, both contiguous basally until about midheight; two densely and strongly setose lobes (**l**) paramedially (Fig. 6O); each half with two higher central pieces with a seminal groove between, frontal piece (a) elongate; long, distally microplumose flagella (f) (Fig. 6K–M); caudal piece (b) subquadrate, membranous, micropapillate frontolaterally, with an apical field of coniform microsetae laterally (Fig. 6L, N).

###### Remark.

The *javanicus*-group is currently comprised of 23 species, including this new species, a fourth in this group to be reported from Laos.

**Figure 8.**
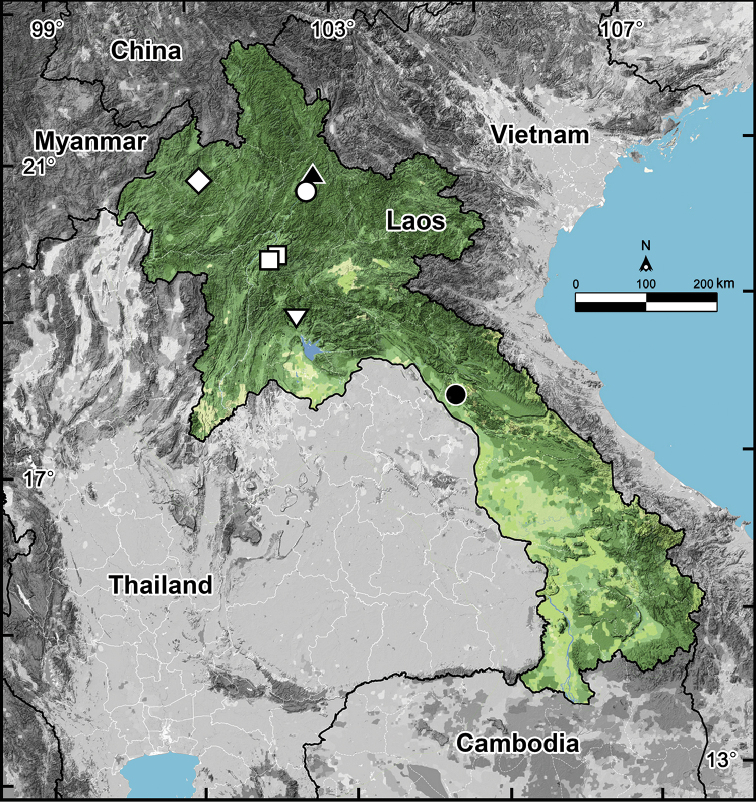
Distribution of *Glyphiulus* species in Laos (6 species): Open diamond: *Glyphiulus semicostulifer* sp. n. Open circle: *Glyphiulus bedosae* Golovatch, Geoffroy, Mauriès & VandenSpiegel, 2007. Filled triangle: *Glyphiulus costulifer* Golovatch, Geoffroy, Mauriès & VandenSpiegel, 2007. Open square: *Glyphiulus subbedosae* sp. n. Inverted open triangle: *Glyphiulus subcostulifer* Golovatch, Geoffroy, Mauriès & VandenSpiegel, 2007. Filled circle: *Glyphiulus percostulifer* Golovatch, Geoffroy, Mauriès & VandenSpiegel, 2007.

#### Key to *Glyphiulus* species known from Laos, chiefly based on male characters

**Table:** 

1	♂ leg 1 very strongly reduced, completely lacking any median structures (Figs 3A, B, 4C)	**2**
–	♂ leg 1 either normal or reduced in size, but then with a pair of paramedian coxal processes (Figs 6A, 7C)	**3**
2	Paraprocts with a row of several strong setae near median marginal ridge (Fig. 2Q); posterior gonopods broadly subquadrate, each half with a plumose apical flagellum (f) (Fig. 3I, J)	***G. subbedosae* sp. n.**
–	Paraprocts with a bare marginal ridge devoid of setae; posterior gonopods narrowly subrectangular	***G. bedosae***
3	Carinotaxy formula of midbody segments 2+I/i+3/3+I/i+2	**4**
–	Carinotaxy formula of midbody segments 2/2+I/i+3/3+I/i+2/2	**5**
4	Carinotaxy formula of collum, I–VI+7a+pc+ma+pc+7a+VI–I, texture of both lateralmost crests unusually micropunctate; ♂ leg 1 with two low, paramedian, contiguous cones; each posterior gonopod with a long and bare flagellum	***G. costulifer***
–	Carinotaxy formula of collum, I+2c+III–VI+5c+6a+pc+ma+pc+6a+5c+VI–III+2c+I, both lateralmost crests as usual, smooth (Fig. 5A–C); ♂ leg 1 with medially contiguous, apically diverging cones (Figs 6A, 7C); each posterior gonopod with a long, distally plumose flagellum	***G. semicostulifer* sp. n.**
5	Coloration entirely pallid, ocelli invisible; adult body up to about 1.0 mm wide; ♂ leg 1 telopodites normal, 5-segmented; apicomedial outgrowths of anterior gonopodal coxosternum especially high and large, telopodite smaller than these apicomedial outgrowths	***G. percostulifer***
–	Coloration usually darker, ocelli always dark and well-discernible; adult body up to about 1.3 mm wide; ♂ leg 1 telopodites reduced in sized, but still 5-segmented; apicomedial outgrowths of anterior gonopodal coxosternum less conspicuous, telopodite as high as these apicomedial outgrowths	***G. subcostulifer***

## Conclusions

Most *Glyphiulus* species in Laos come from caves or surrounding areas, except for *G. subbedosae* sp. n. found epigeically near waterfalls. Several of the cave species show troglomorphic traits such as an unpigmented tegument and ocellaria (if any), combined with elongated antennae and legs (Golovatch et al. 2011a). The above two new species, however, are pigmented and have short antennae and legs, while the epigean *G. subbedosae* sp. n. presents a distinct colour pattern. Such characters are rather evidence of the cave-dweller *G. semicostulifer* sp. n. being only a troglophile likely to occur also outside caves. Usually only a single cambalopsid species is found per cave. The single exception known so far concerns two *Plusioglyphiulus* species, *P. bedosae* Golovatch, Geoffroy, Mauriès & VandenSpiegel, 2009 and *P. pallidior* Golovatch, Geoffroy, Mauriès & VandenSpiegel, 2009, coexisting in the same cave in Kalimantan, Borneo, Indonesia, but both these species differ so strikingly in body size that this alone strongly suggests niche segregation (Golovatch et al. 2009).

The diplopods of Laos are still poorly known, with only a small fraction of their diversity being assessed. There is little doubt that, with further progress in the study of the millipede fauna of Laos, both epigean and cavernicolous, many more novelties are to be expected. As regards the Cambalopsidae alone, we seem to have only touched the tip of the iceberg (Golovatch et al. 2007a).

## Supplementary Material

XML Treatment for Glyphiulus subbedosae

XML Treatment for Glyphiulus semicostulifer
